# Behaviourally Mediated Phenotypic Selection in a Disturbed Coral Reef Environment

**DOI:** 10.1371/journal.pone.0007096

**Published:** 2009-09-18

**Authors:** Mark I. McCormick

**Affiliations:** ARC Centre of Excellence for Coral Reef Studies and School of Marine and Tropical Biology, James Cook University, Townsville, Queensland, Australia; Northeastern University, United States of America

## Abstract

Natural and anthropogenic disturbances are leading to changes in the nature of many habitats globally, and the magnitude and frequency of these perturbations are predicted to increase under climate change. Globally coral reefs are one of the most vulnerable ecosystems to climate change. Fishes often show relatively rapid declines in abundance when corals become stressed and die, but the processes responsible are largely unknown. This study explored the mechanism by which coral bleaching may influence the levels and selective nature of mortality on a juvenile damselfish, *Pomacentrus amboinensis*, which associates with hard coral. Recently settled fish had a low propensity to migrate small distances (40 cm) between habitat patches, even when densities were elevated to their natural maximum. Intraspecific interactions and space use differ among three habitats: live hard coral, bleached coral and dead algal-covered coral. Large fish pushed smaller fish further from the shelter of bleached and dead coral thereby exposing smaller fish to higher mortality than experienced on healthy coral. Small recruits suffered higher mortality than large recruits on bleached and dead coral. Mortality was not size selective on live coral. Survival was 3 times as high on live coral as on either bleached or dead coral. Subtle behavioural interactions between fish and their habitats influence the fundamental link between life history stages, the distribution of phenotypic traits in the local population and potentially the evolution of life history strategies.

## Introduction

Habitat change through natural or anthropogenic causes is implicated as the greatest threat to global biodiversity [1]–[3]. As habitats alter they become more or less favourable and the way organisms use space changes [4], [5]. These shifts in the use of space may influence vulnerability to predators and alter the selective regime imposed on the community [6]. If this selection is directional then habitat change can influence the evolutionary trajectories of affected species by altering the distribution of traits in the surviving population [7].

Factors that influence an individual's probability of survival depend upon the interaction between the predator and prey, and the context within which the interaction occurs. If the context makes the prey more vulnerable, then this context may be the primary source of inequality between prey individuals. For instance, patchy habitat features may make one prey more visible than another, or alternatively interactions between prey may decrease individual vigilance or place certain individuals in closer proximity to a foraging predator. Previous studies on a wide range of taxa have found prey selection regimes to be strongly influenced by habitat [6], [8], [9] and social environment [10], [11]. The probability of loss is also affected by attributes of the prey (e.g. body size) that lead to differential performance in an interaction with a predator possessing an appropriate selection profile [12]. Little is known of how habitat affects the social interactions among prey individuals that shape differential vulnerability, and ultimately the selective nature of mortality.

Coral reefs are one of our most diverse ecosystems, but they are also under the greatest threat through changes in habitat composition as a result of carbon dioxide induced climate change [13], [14]. Global sea surface temperatures are predicted to increase by 1.1°C and 6.4°C by 2100 depending upon various future CO_2_ emissions scenarios [14]. Exposure of corals to temperatures that are elevated more than a few degrees above the long term average can lead to expulsion of their symbiotic zooxanthellae (known as bleaching) and may lead to death [15]. Bleaching has already substantially reduced global coral cover [13], [16] and is predicted to intensify with warming temperatures [17].

This change in live coral cover is accompanied by a change in the fish community associated with coral, but little is known of the processes and mechanisms underlying these changes in community composition. While only 9–11% of fish species are coral dependent [18], [19], a large proportion of the diverse tropical fish communities (upwards of 60%) rely on live hard coral for settlement [19], [20] and show declines paralleling reductions in hard coral cover [19], [21]. The death of corals caused by bleaching is often patchily distributed on scales within metres [22], [23]; one colony will die, while another colony of the same species next to it will appear healthy. The loss of corals and patchiness of surviving colonies suggests that settling fish will become concentrated on the remaining live corals. Enhanced density dependence on the remaining live coral may lead to an accentuation of the importance of processes such as competition [24] in mediating local mortality levels and may alter the selective nature of this loss.

Settlement for fishes with dispersive larvae is a critical period, as it is for all organisms with complex life histories such as amphibians and many invertebrates [25], [26]. Mortality during settlement is both extremely high and often selective for individual attributes [10], [27], [28]. Size advantages are often important immediately upon settlement and can influence the suite of co-varying traits that carryover to later life stages [29]–[31]. It is unclear how coral bleaching will influence the nature of selective mortality, or how characteristics of individuals at settlement may influence the distribution of traits among habitats.

The present paper explores the behavioural mechanism underlying mortality and selective loss of individuals from a coral habitat disturbed by bleaching. The study focuses on the ambon damselfish *Pomacentrus amboinensis*, who preferentially settle on live coral [20], [32], but are found on a variety of habitats as juveniles and adults. Understanding how *P. amboinensis* responds to bleaching may elucidate the mechanisms of change for the large number non-coral obligate species that show declines in response to coral degradation [21]. A series of three experiments address the questions: How site attached are juvenile fish when they settle to a patchily disturbed environment? Does high density increase the likelihood of individuals moving to a bleached patch and can fish size predict an individual's likelihood of migration? How does habitat influence behaviour and what is the role of fish size? How does habitat influence fish mortality? Findings suggest that the way peers interact and use space is noticeably altered by habitat change and this directly influences not only mortality levels but the direction and intensity of size selective loss.

## Materials and Methods

### Study species

The damselfish *Pomacentrus amboinensis* is common on coral reefs of the Indo-Pacific. Males and females are strongly site attached and often live in a discrete group. *P. amboinensis* has a pelagic larval duration of 15–23 days and settles at 10.3–15.1 mm standard length with its juvenile body plan largely complete [33]. Although *P. amboinensis* settle to a wide variety of habitats on reefs of the northern Great Barrier Reef they are found in highest densities associated with mixed live coral, rubble and sand areas on the shallow reef base or reef slope. A tagging study of 295 newly settled fish on the continuous reef edge found that fish moved little over the first 3 months after settlement (mean = 0.63 m [34]). A recent study showed that *P. amboinensis* who had newly metamorphosed in light traps displayed a dramatic and statistically significant preference for live coral (*Pocillopora damicornis*) over bleached, dead coral or sand habitats in selection trials conducted in 500 l circular tanks [35]. Studies of this species also suggest high levels of mortality are typical within the first 24 h on the reef, with values of up to 98% mortality being recorded (mean ∼50%) [9], [10]. Interestingly, despite this high mortality, McCormick and Gagliano (in press [36]) showed that for *P. amboinensis* there is a relationship between the size of the otolith at hatching and who changes sex to become the dominant male later in life; that is, early life history somehow pro-rates subsequent individual success. This link stresses the importance of carry-over effects between life stages to the fundamental population dynamics of this species.

Newly settled reef fishes tend to be site attached and are subject to an array of resident and transient predators [see 9,37]. Predators can be seen striking at and occasionally capturing recently settled and juvenile reef fishes during the summer recruitment period.

A previous study that undertook behavioural observations on 10 fish over 20 consecutive 1 min periods found that a 3 min observation period was sufficient to obtain a representative quantification of behaviour for *P. amboinensis* (Mero and McCormick unpublished data; also see [38]).

### Ethics statement

This research was undertaken with approval of the James Cook University (JCU) animal ethics committee (JCU ethics permit: A112) under the JCU animal ethics guidelines.

### Experimental design

The present study was conducted at the base of a shallow reef at Lizard Island (14°38′S, 145°28′E), northern Great Barrier Reef, Australia, during October to December 2008. In overview, the study involved three separate experiments. The first experiment measured size-related mortality trajectories on three different habitats. Large and small newly metamorphosed *P. amboinensis* were placed in pairs onto either live healthy *Pocillopora damicornis* (a bushy hard coral); thermally bleached *P. damicornis* (see protocol below); or dead *P. damicornis*. The dead coral was structurally intact and with small levels of algal growth and fouling invertebrates. Patches were spaced to preclude fish migration between patches and observations were conducted for up to 140 h. The second experiment examined movement between habitat patches and the influence of fish size. Here, the first experiment was repeated, but this time the three different habitat types were placed in close proximity to one another to allow fish migration. Fish were monitored for ∼48 h and their behaviour was quantified in detail. The third experiment examined whether high densities of fish on small patches of live coral promoted movement to nearby bleached or dead patches, and whether that movement was related to fish size. This experiment had the same spatial design of clusters of three habitat types in close proximity, but placed 6 tagged fish of a size range on the live coral patch. Movement and limited aspects of behaviour were recorded for 48 h.

#### Experiment 1: Habitat and size related persistence

Light traps were used to collect *P. amboinensis* at the end of their larval phase. Fish were placed into an aquarium with aerated flowing seawater. Fish were kept for 24 h and fed newly hatched *Artemia* sp. twice per day *ad libitum* to allow recovery from (or acclimation to) the stress of capture, prior to sizing and tagging. There was minimal mortality during this time. Individual fish were placed into clip-seal plastic bags containing aerated seawater and measured with digital calipers (±0.1 mm). Fish were paired, such that one individual (‘large’ individual) was 0.8–1.0 mm greater in standard length than the other (‘small’) (see supporting information Figure S1). To enable individual identification fish were tagged through the plastic bag with either a red or blue subcutaneous fluorescent elastomer tattoo using a 27-gauge hypodermic needle (as per 39). This left a 1.5–2 mm long stripe of colour, which was visible under the scales. Colours were alternated between large and small fish among replicates to avoid the possible bias of predators selecting prey based on tag colour. Previous studies have found no evidence of this selection (T. Holmes unpublished data). Tagging with a single elastomer tattoo has been found to have no influence on the mortality or growth of this species [39]. Behaviour observations in the field previously [e.g. 9], [10], [12], [40], [41] and in the present study have indicated no size-dependent adverse affect of tagging.

Size-paired fish were transported to the study site in 2 l plastic bags of aerated seawater, and then released onto small patches (20×10×15 cm) of one of the three substrata: healthy *P. damicornis* (a bushy hard coral); thermally bleached *P. damicornis* (see below); dead *P. damicornis*. Habitat patches were about 4 m from the hard reef edge on sand and organized in a row 5 m apart to prevent migration between patches. All fish and mobile invertebrates were removed from the substrata prior to the commencement of a trial. A small wire cage (∼30×30×30 cm, 6 mm mesh size) was placed over each patch for 30–40 min to allow the tagged fish to acclimate to their new surroundings while being protected from predators. Fish were released onto the reefs between 10:00 and 10:30 h. Survival of tagged fish was monitored 2–3 times per day (morning, mid-day, evening) by visual census (occasionally the mid-day census was not undertaken). When one or both individuals were missing the adjacent area (within 3 m of the release site) was searched to determine if the fish had simply migrated. During the study period, densities of up to six newly settled *P. amboinensis* were found to naturally occur on dead and live coral habitats of the size used in this study. Monitoring finished when both tagged individuals were lost from the sites, or was terminated due to the end of a field trip.

#### Experiment 2: Inter-habitat movement

Clusters of the three different habitat types were constructed to examine the importance of movement between patches and the social dynamics that may underlie mortality of individuals within a group. Clusters were composed of one small coral head (20×10×15 cm) of each of three coral habitat types 0.4 m from one another: healthy Pocillopora damicornis, bleached P. damicornis, and dead P. damicornis.

In a similar methodology to above, *P. amboinensis* caught in a light trap were kept for 24 h, measured with calipers, tagged with one of six different colours and size matched for a 0.8–1 mm difference in SL (as above). Pairs were then transported out to the field in a labeled plastic bag at 10:00–11:00 h. One pair was placed on each of the three substrata per cluster. Patches were visually obscured from one another by plastic barriers during a 30 min acclimation period, during which time fish were also enclosed within a 6 mm mesh cage to prevent predation (as above). After the acclimation period the barriers and then the cages were removed. Fifteen minutes after release the behaviour of all fish within the set of three patches was quantified in detail (3 min each; see below), and this was repeated over 48 h at ∼16:00 and ∼11:00 h. Mortality and movement between patches was also assessed at this time.

#### Experiment 3: Migration at high densities

To determine the likelihood of a fish migrating to a bleached or dead coral when the density of fish on a live coral patch is very high, fish were stocked in high density onto live coral patches and given the choice of dead or bleached coral patches nearby. The configuration and composition of patches was exactly the same as above (experiment 2), with the exception that only the healthy *Pocillopora* coral head was stocked with 6 newly settled *P. amboinensis*. Fish were once again tagged for individual recognition and they were measured as above prior to release. Acclimation procedure was the same as experiment 2 and the location of fish were monitored over 48 hours as above. Experiment 3 was conducted 2 weeks after experiment 2 and at the same location.

### Fish behaviour

In experiment 2 the behaviour of fish on the three patch types within each cluster was assessed over 3 min periods. Behaviour of the fish was assessed by a scuba diver positioned 1.5 m away from the patch. A magnifying glass (4x) aided the assessment of bite rates and space use over the 3 min focal animal sampling period. Six aspects of activity and behaviour were assessed: a) total distance moved; b) distance ventured from the habitat patch (categorized as % of time spent within 0, 2, 5 or 10 cm away from the patch); c) height above substratum (categorized as % of the time spent within the bottom, middle or third of the patch); d) number of fin displays; e) the number of chases or bites; f) number of avoidance episodes in response to a conspecific; g) boldness (recorded as a variable on a scale from 0 to 3 at 0.5 increments, where: 0 is hiding in hole and seldom emerging; 1 is retreating to hole when scared and taking more than 5 sec to re-emerge, weakly or tentatively striking at food; 2 is shying to shelter of patch when scared but quickly emerging, purposeful strikes at food; and 3 is not hiding when scared, exploring around the coral patch, and striking aggressively at food). At the end of the 3 min observation period, the fish was approached with a finger and the fish's reaction and latency to emerge from shelter was taken into account in the assessment of boldness. Two additional variables were devised from these variables to summarise information and reduce the number of variables that were required in analyses. Relative height on the patch was summarized as a cumulative proportion of the time spent at varying heights over the 3 min observation period, with the top of the patch taken as height of 1, mid-patch a height of 0.5, and bottom a height of 0. An aggression index was also created by adding the number of displays to the product of three times the number of chases/bites and then subtracting the number of avoidance events. A weighting factor of three was used in conjunction with the chases/bites as the influence of this behaviour on the spatial distribution of the recipients appeared to be many times greater than their response to displays.

In experiment 3, the dominance status of the individuals within each pair was also categorised from the ∼10 min observation period as dominant or subordinate, based on the number of displays, chases and avoidances.

### Coral bleaching

Bleaching (the loss of zooxanthellae) was induced by placing *Pocillopora damicornis* colonies in 500 l seawater aquaria and raising the temperature incrementally over 48 h from ambient (28°C) to a sustained maximum of 32°C for 9 to 10 days. Aquaria were constantly aerated, flow maintained using 2 1220 l.hr^−1^ powerheads and heated with two 300 W aquarium baton heaters under very low light levels. After colonies were visibly bleached, water temperature was lowered to ambient incrementally over 48 h. This protocol resulted in a live coral with few or no zooxanthellae that would stay bleached in the field for up to 2 months. Many ended up regaining zooxanthellae over a 6 to 8 week period.

### Analyses

To examine the differences in behaviour between large and small fish among the three habitat treatments repeated measures ANOVAs (RMANOVAs) were conducted. In these analyses fish size (large or small) was used as the variable on which the repeated measure was undertaken (effectively pairing the observations; [42]). As there are only two levels of size the assumption of sphericity was irrelevant. Assumptions of normality and homogeneity of variance was examined using residual analysis. Tukey's HSD tests were undertaken after RMANOVA to explore the nature of any significant differences found among more than 2 means. Trends were tested for data collected 15–30 min after cage removal. To determine whether the spacing pattern between large and small fish differed among habitats after cage removal, a one-factor ANOVA was undertaken to test the equality of distances between fish. To estimate this variable the difference between the distance from the patch for the largest fish was subtracted from the smallest fish. Data were log_10_(x+1) transformed.

Survival (up to 165 h) between large and small fish on the three substrata was compared using Survival Analysis (Statistica 8.0). Survival curves of each fish size and substrata were calculated and plotted using the Kaplan–Meier product–limit method. The Kaplan–Meier method is a non-parametric estimator of survival that incorporates incomplete observations, such as those cases where censuses had to be terminated on trials prior to their completion due to time limitations of a field trip. Projected survival were compared between the three substrata using a Chi-square statistic, while differences in survival between large and small fish were compared using a Cox-F statistic.

## Results

### Movement

When two fish that differed by 0.8–1 mm SL were placed on each of the 63 habitat patches (representing 21 clusters of 3 habitats), 15.1% moved from their original placement to one of the other habitats in the cluster (i.e., 19 out of 126 fish moved). The original habitat they were on did not influence whether they moved or not, with equal numbers moving from each habitat (healthy coral, 14.2%; bleached coral, 16.7%; dead coral, 14.2%). There was no obvious preference for the habitat to which the 19 fish moved (7 moved to live coral; 7 to bleached coral; 5 to dead). Twelve out of 19 fish that moved habitats were the smaller, subordinate individual of the pair. All the smallest individuals (6 fish) within the cluster moved habitats more than once over the census period.

When the density of fish on live coral was boosted to the upper limit of natural densities (6 per patch) 12 out of 78 fish (15.4%) moved to the dead or bleached patch within the cluster; a similar figure to the frequency of movement when two fish were placed on each patch within a cluster (15.1% above). Seven of the 12 fish moved to bleached coral, and the remaining 5 went to the dead coral patch. All fish that moved were the smallest or second smallest fish allocated to the clusters.

### Behaviour

**Figure 1:**
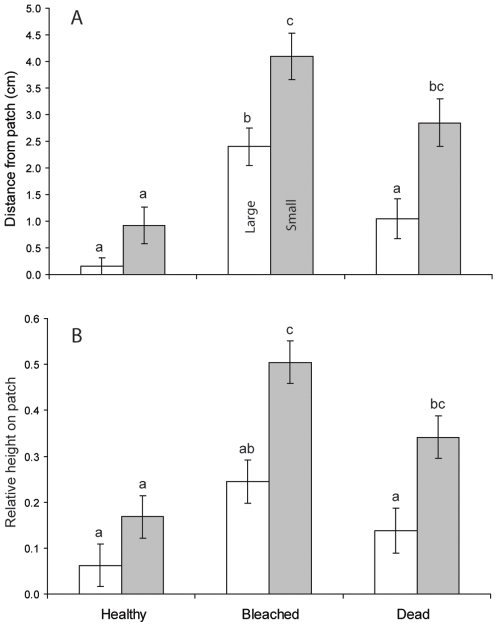
Influence of coral degradation on fish vulnerability. Spatial patterns of large and small *Pomacentrus amboinensis* (large is 0.8 to 1.0 mm larger than small) placed in pairs (one large, one small) onto patches of three hard coral habitat types (healthy, bleached and dead *Pocillopora damicornis*). (*A*) mean horizontal distance from the patch reef recorded 30 min after release, and (*B*) mean relative height on the patch reef (a value of 1 represents 100% of time spent on top of patch; 0, 100% time at the base). Letters above the bars represent Tukey's HSD groups. Errors are standard errors.

The behaviour of large and small fish differed among the three habitats. There was a significant interaction between fish size and habitat that influenced their distance from the patch reefs 15–30 min after removal of the cage (RMANOVA: Treatment x Size, F_2,60_ = 3.288, p<0.04; Treatment, F_2,60_ = 10.937, p<0.0001; Size, F_2,60_ = 75.984, p<0.0001). Large fish were closer to shelter than the smaller fish on bleached and dead coral habitats (Tukey's HSD tests), while on healthy corals both stayed close to shelter (Figure 1A). Large fish were significantly further from shelter on bleached coral, where both large and small fish were over 2 cm from the edge of the coral (Figure 1A).

There was also a significant interaction between fish size and habitat that influenced the relative height of large and small fish on the habitat patches (RMANOVA: Treatment x Size: F_2,57_ = 3.330, p = 0.04; Treatment, F_2,57_ = 12.420, p<0.0001; Size, F_1,57_ = 45.875, p<0.0001). Large fish were closer to the base of the patch than the smaller fish on bleached and dead coral habitats (Tukey's HSD tests), while on healthy corals both stayed close to the base (Figure 1B).

The distance between smallest and largest fish 15 min after cage removal also differed among habitats (log_10_(x+1) transformed; F_2,50_ = 6.174, p = 0.004), with the distance between fish within a pair being significantly smaller on healthy coral than on the other two habitats (as determined by Tukey's HSD tests).

Large individuals of the pair were the most aggressive individuals (mean±se: large 7.18±0.92, small −1.51±0.9; RMANOVA, Size: F_1,57_ = 30.296, p<0.0001), regardless of habitat (RMANOVA, Treatment: F_2,57_ = 0.146, p = 0.864; Treatment x Size: F_2,57_ = 0.660, p = 0.521). Large individuals of the pair were also the boldest individuals (large 1.7±0.09, small 1.43±0.09; RMANOVA, Size: F_1,56_ = 5.498, p = 0.022), regardless of habitat (RMANOVA, Treatment: F_2,56_ = 1.705, p = 0.191; Treatment x Size: F_2,56_ = 0.103, p = 0.903).

**Figure 2:**
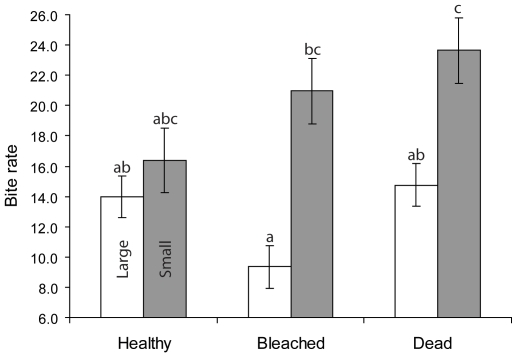
Influence of coral degradation and fish size on foraging rates. Comparison of bite rates (per 3 min) of large and small newly-settled *Pomacentrus amboinensis* on three different habitat patch types. Errors are standard errors.

The bite rates of large and small fish when in pairs differed among habitats (RMANOVA: Size x Habitat: F_2,58_ = 17.397, p<0.0001; Treatment, F_2,58_ = 1.842, p = 0.168; Size, F_1,58_ = 135.381, p<0.0001; Figure 2). Large and small fish displayed the same bite rates when on healthy coral, but small fish had the highest bite rates on bleached and dead coral (Figure 2). The largest difference between bite rates of large and small fish was found on bleached corals (Figure 2).

### Mortality

**Figure 3:**
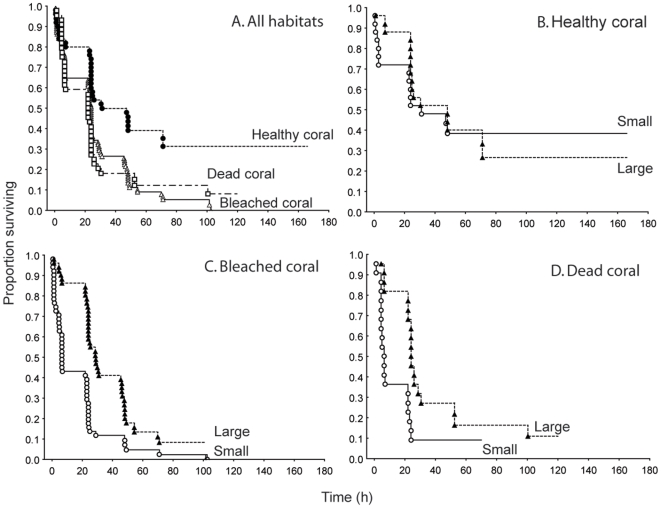
Comparison of fish size-specific survival on 3 coral habitats. Survival trajectories of *Pomacentrus amboinensis* regardless of size on three habitats (*A*), and then of large (triangles) and small (circles) fish that were settled onto (*B*) healthy live coral, (*C*) thermally bleached coral, and (*D*) dead coral.

Habitat type influenced levels of mortality of *P. amboinensis* (Chi-square = 12.671, df = 2, p = 0.0018; Figure 3A). Survival was highest on healthy coral with 32% surviving 70 h after settlement (Figure 3A). Survival was equally low on bleached and dead coral (8 and 14% respectively surviving 70 h; Figure 3A).

On healthy coral, small and large fish within the pairs survived equally well (Cox's F-Test, F_34,30_ = 1.029, p = 0.471; Figure 3B). On bleached coral large fish survived better than small fish (Cox's F-Test, F_92,100_ = 2.273, p<0.0001; Figure 3C). Large fish also survived longer than small fish on dead coral skeletons (Cox's F-Test, F_38,40_ = 2.2607, p<0.0001; Figure 3D).

## Discussion

Unfortunately for fish communities, the high water temperatures and light intensities that promote coral bleaching peak during the summer months [43], when the majority of fishes reproduce and settle. This means bleaching will most likely occur when many fishes are at their most vulnerable: when they are small, settling naïve to reef based predators, and subject to high predation pressure. Findings of the present study suggest that the change from live coral, through bleached to algal covered dead coral, directly induced changes in the behaviour of individuals at settlement, which enhanced not only the intensity of mortality, but also altered the phenotypic selection on important traits such as fish size. Even when fishes are ecologically versatile and will use settlement substrata other than live coral, such as the present species that is known to settle onto rubble [20], [32], the present study indicates that levels of mortality are likely to be higher than they would have been on live coral. The loss documented here was rapid, occurring hours after settlement, and therefore would be largely undetected by monitoring surveys designed to detect broad scale changes in community composition and relative abundance.

Movement of fish was minimal after settlement even when distances were small and densities were close to the maximum that naturally occurred. Some individuals moved to explore other patches, but came back to their original patch. This stresses the importance of their initial choice in influencing their fate. High site fidelity in recently settled fishes is a common finding among bottom-associated coral reef fishes [44]. Evidence suggests that if fish are on corals that subsequently bleach they are unlikely to migrate to live coral, despite exhibiting higher survival on live coral. Bleached coral may act as an ecological trap [45] for species that are attracted to it, migrate to it, or end up on it through the bleaching process; it provides a settlement stimulus which is no longer optimal and does not support viable populations [46]. Interestingly, those fish that moved most often were the smallest individuals within the experimental groups that were subject of aggression from larger individuals. Larger fish, even when on bleached coral, defended their habitat patch and were less likely to move to a different habitat patch. This means that while small individuals may initially be exposed to higher predation by nature of their subordinate status, they may still have greater overall survival if they successfully move from bleached or dead coral, where survival was lowest in the longer term. The amount of movement of this kind was moderate (∼15% of small fish) so could ameliorate some of the directional selection imposed on small fishes in degraded habitats.

**Figure 4:**
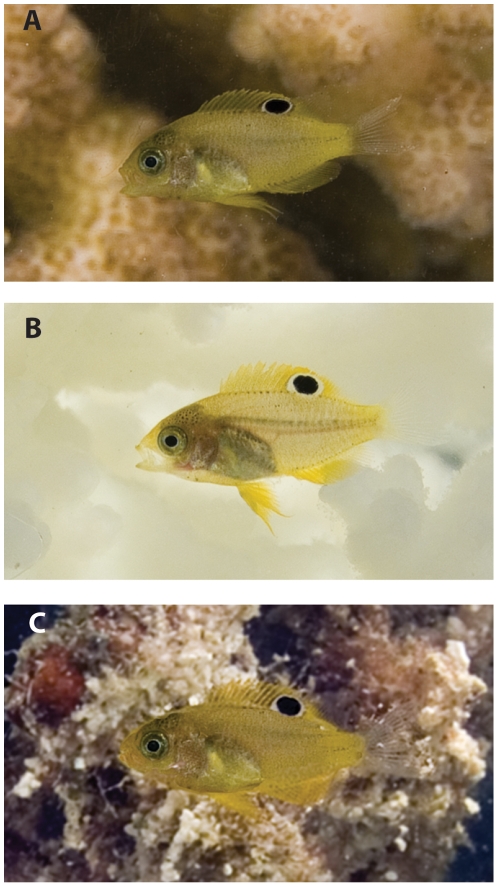
Typical contrast of recently settled *Pomacentrus amboinensis* against 3 common habitat backgrounds: a) live *Pocillopora damicornis*, b) thermally bleached *P. damicornis* and c) dead *P. damicornis*.

Behaviour immediately after settlement differed depending upon whether the coral was in a healthy live state, bleached or dead. On bleached coral fish were higher from the base of the patch and further out from the coral than when on live coral. Fish on dead coral displayed a use of space intermediate between these two extremes. Large fish were closer to the base and in closer proximity to the coral than smaller fish. There are a number of possible non-exclusive reasons for these habitat-related differences in space use. Many marine predators have visual systems that rely on contrasting colouration to detect prey [47]. Yellow fish, such as *P. amboinensis*, will be effectively camouflaged if they can distance themselves from the monochrome white bleached background to be seen against the upwelling yellow/brown light from the sand [48], [49] (Figure 4). A study of the spectral qualities of fish and their habitats found that yellow fish are effectively camouflaged against an average live coral reef because they have very similar colour reflectance [49]. Similar reflectance is emitted from yellow and brown sand on shallow reefs (Marshall pers. comm.). Since most predators on recruits are mid-water (e.g. sling-jaw wrasse, coral trout) and bottom dwelling (e.g. cods and lizardfish) species, this avoidance strategy is most effective when prey are close to the sand, within the shadow of the coral. This appears to be the strategy adopted by the largest, dominant fish on bleached coral. On live coral, yellow fish will be best camouflaged when close to the mottled colour of the live coral [48], [49]; the position adopted by both large and small fish on live coral. There should be strong selective pressure to minimize risk of predation when most vulnerable, such as when newly settled. If behaviour alters distribution to maximize camouflage (minimize risk) against their background then it could be expected that fish with different colouration would have differing behaviour strategies to minimize predation risk by positioning themselves in the position of lowest average contrast [49]. This may mean that habitat-related rates of mortality and the nature of selection (directional, disruptive or stabilizing) will be species specific.

Alternatively, though not exclusive of the first argument, bleached and algal/invertebrate covered dead coral may emit olfactory cues that are repugnant or confusing to settling fish. A recent study using cafeteria-style choice trials that excluded visual stimuli and promoted olfactory signals found that three damselfish species consistently avoided bleached coral [35]. Late stage larval and newly metamorphosed fishes typically have well developed olfactory systems well before metamorphosis [50] and are known to use their sense of smell to navigate [51], [52] and detect conspecifics or habitat during settlement [53], [54]. During bleaching the symbiotic zooxanthellae die, become necrotic and are expelled [55]; a process that is likely to leave an olfactory signal that may be detectable by fishes for some time afterwards [56]. Whatever the reason, it is interesting that the large, dominant fish also move further from the bleached patch suggesting that something associated with the loss of zooxanthellae from the coral is causing the shift in behaviour.

This paper underscores the importance of behavioural interactions around the time of settlement in influencing levels of mortality on different coral habitats. Even though differences in the sizes of newly settled fish were small (∼6–8% difference), the larger fish of the pair was always dominant, exhibiting higher boldness and aggression regardless of their absolute size. The estimated distance between fish was higher on bleached and dead coral than on live coral. This suggests that large fish are either more aggressive on degraded corals or the signals produced by the dominant or received by the subordinate were more effective. Analysis showed no significant difference in aggression with habitat, however there was approximately 48% greater difference in mean aggression levels between large and small fish on bleached coral. It is not uncommon to find that intra-peer aggression varies with habitat [57], [58] since subtle differences in habitat influence predation risk [59] and the potential importance of specific areas as key refuge sites. This study is the first to underscore the important role played by behavioural interactions between cohort members immediately after settlement in driving the dynamics of post-settlement mortality, phenotypic selection profiles and post-settlement distribution patterns.

Acquisition of the spatial position of lowest risk by the large individuals in a pair traded-off immediately against feeding rate (and possibly growth) in the two degraded habitats. Large fish on dead and bleached coral stayed closer to the base of the reef and closer to shelter, and had a lower feeding rate than small fish that were positioned higher in the water column and further from the patch. Since planktivorous fishes usually feed on items well below their gape size, fish that are further into the current have access to more and larger food items than fish down-current. Other studies have found that planktivorous fish that were positioned higher and further out from their habitats had higher feeding rates and ingest higher quality food [60], [61]. In these studies, this position was secured by dominant individuals, not subordinates. Large, dominant individuals often monopolise the best or greatest amount of prey in animals with strong dominance control (e.g. lions [62], chimpanzees [63], brown bears [64]). Obviously, since mortality is highest on small fish, the strategy of being exposed with a high feeding rate is suboptimal at this vulnerable life stage.

Hard coral loss through degradation, such as bleaching, disease or corallivore predation, leads to a widespread decline in the abundance and species diversity of fishes [65]. In a recent meta-analysis, Wilson *et al.* (2006 [21]) found that 62% of fish species examined declined following a 10% or greater loss in coral cover. Coral dwellers and feeders were most vulnerable, but many invertebrate feeders and planktivores also showed marked declines. Rapid changes are attributed to the negative impacts of coral degradation on settlement levels in fishes [19], [66]. To compound matters, the present study found that mortality after settlement was much higher on non-live coral habitat patches, and movement to alternative habitat patches was limited even for an ecologically versatile species. An unknown in this dynamic landscape of population processes is how changes in phenotypic selection will impact the range of phenotypic and behavioural traits entering the reproductive life stages. Depending upon the consistency of this selective regime [67], [68], which appears to be mediated by the behaviour of the prey, rather than that the selective profile of the predator, these shifts in the nature of mortality may have impacts on the fundamental links between life history stages and the evolution of life history strategies [69].

## Supporting Information

Figure S1 Overall size frequency distributions of newly metamorphosed Pomacentrus amboinensis placed in pairs onto habitat patches to examine habitat-related size selection. Individuals with each pair differed in size by 0.8–1 mm standard length.
(3.48 MB TIF)
