# Supplementary figures and images for: Behaviourally Mediated Phenotypic Selection in a Disturbed Coral Reef Environment
Source: PLoS One. 2009 Sep 18;4(9):e7096. doi: 10.1371/journal.pone.0007096 (PMC2740825; doi:10.1371/journal.pone.0007096)

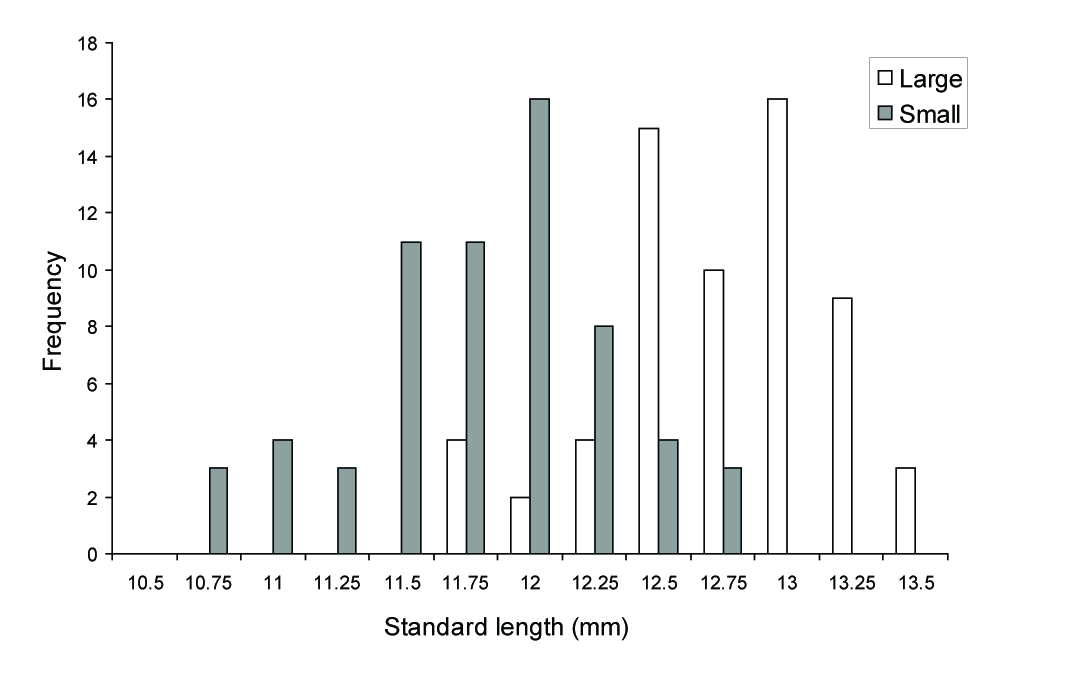

Supplement: Figure S1 — Overall size frequency distributions of newly metamorphosed Pomacentrus amboinensis placed in pairs onto habitat patches to examine habitat-related size selection. Individuals with each pair differed in size by 0.8–1 mm standard length. (3.48 MB TIF) [file pone.0007096.s001.tif]
